# MicroRNA-30a increases tight junction protein expression to suppress the epithelial-mesenchymal transition and metastasis by targeting Slug in breast cancer

**DOI:** 10.18632/oncotarget.7656

**Published:** 2016-02-24

**Authors:** Chia-Wei Chang, Jyh-Cherng Yu, Yi-Hsien Hsieh, Chung-Chin Yao, Jui-I Chao, Po-Ming Chen, Hsiao-Yen Hsieh, Chia-Ni Hsiung, Hou-Wei Chu, Chen-Yang Shen, Chun-Wen Cheng

**Affiliations:** ^1^ Institute of Biochemistry, Microbiology and Immunology, Chung Shan Medical University, Taichung, Taiwan; ^2^ Department of Surgery, Tri-Service General Hospital, National Defense Medical Center, Taipei, Taiwan; ^3^ Department of Surgery, Chung Shan Medical University Hospital, Taichung, Taiwan; ^4^ Department of Biological Science and Technology, National Chiao Tung University, Hsinchu, Taiwan; ^5^ Institute of Biomedical Sciences, Academia Sinica, Taipei, Taiwan; ^6^ College of Public Health, China Medical University, Taichung, Taiwan; ^7^ Clinical Laboratory, Chung Shan Medical University Hospital, Taichung, Taiwan

**Keywords:** breast cancer metastasis, EMT, miR-30a, slug, claudin

## Abstract

The epithelial-to-mesenchymal (EMT) transition is a prerequisite for conferring metastatic potential during tumor progression. microRNA-30a (miR-30a) expression was significantly lower in aggressive breast cancer cell lines compared with non-invasive breast cancer and non-malignant mammary epithelial cell lines. In contrast, miR-30a overexpression reversed the mesenchymal appearance of cancer cells to result in a cobblestone-like epithelial phenotype. We identified Slug, one of the master regulators of EMT, as a target of miR-30a using *in silico* prediction. Reporter assays indicated that miR-30a could bind to the 3′-untranslted region of *Slug* mRNA. Furthermore, we linked miR-30a to increased expression of claudins, a family of tight junction transmembrane proteins. An interaction between Slug and E-box in the claudin promoter sequences was reduced upon miR-30a overexpression, further leading to reduction of filopodia formation and decreased invasiveness/metastasis capabilities of breast cancer cells. Consistently, delivery of miR-30a in xenografted mice decreased tumor invasion and migration. In patients with breast cancer, a significantly elevated risk of the miR-30a^low^/*CLDN2*^low^/*FSCN*^high^ genotype was observed, linking to a phenotypic manifestation of larger tumor size, lymph node metastasis, and advanced tumor stage among patients. In conclusion, the miR-30a/Slug axis inhibits mesenchymal tumor development by interfering with metastatic cancer cell programming and may be a potential target for therapy in breast cancer.

## INTRODUCTION

The epithelial-to-mesenchymal transition (EMT) enables tumor cells to transiently lose their epithelial features—including the loss of apico-basal polarity and disassembly of tight and adherent junctions—and acquire mesenchymal traits that lead to invasion, metastasis, and resistance to chemotherapy [1, 2]. EMT is characterized by frequent, temporal, and heterogeneous changes in cellular phenotype that cannot be exclusively attributed to rigid and irreversible genetic alterations [3, 4]. Instead, the disruption of normal epigenetic mechanisms provides such essential flexibility, and among epigenetic mechanisms within cancer cells, post-transcriptional mechanisms involving microRNAs (miRNAs) are important in this regard.

miRNAs, which are non-coding RNAs of an average length of 22 nt, bind to the 3′-untranslated region (3′-UTR) of mRNAs with less-than-perfect complementarity, which results in degradation of the mRNA or repression of its translation [5]. A growing body of research has indicated that cancer cells acquire the ability to invade and disseminate via the action of dysregulated miRNAs that enhance EMT progression [6, 7] and confer a selective advantage during clonal evolution [8, 9]. Our previous breast cancer study revealed that miR-30a inhibits the invasion and migration of Hs578T and MDA-MB-231 breast cancer cells *in vitro* [10]. Because these two cell lines are intrinsically deficient in expression of E-cadherin [11], which is a key protein contributing to cell-cell adhesion, we hypothesized that miR-30a targets other mRNAs involved in regulating EMT.

The expansion of a tumor cell population upon overexpression of Snail family members is a prerequisite for EMT [2]. In addition, the regulator Snail (*SNAI1*), which mediates EMT activation for metastatic dissemination of cancer cells from the primary tumor, is targeted by miR-30a [12]. Slug (*Slug*) belongs to the Snail family and also triggers EMT during tumor progression [13]. We thus hypothesized that miR-30a binds to the 3′-UTR of *Slug* mRNA to inhibit EMT-driven invasion and migration in breast cancer. To test this hypothesis, we used an *in vitro* model of the mesenchymal-to-epithelial transition (MET) that is regulated by the miR-30a/Slug axis. The decrease in Slug levels by miR-30a in invasive breast cancer cells resulted in a transformation to a cobblestone-like epithelial phenotype, and ectopic administration of miR-30a led to increased claudin expression, which is transcriptionally inactivated by Slug [14]. Furthermore, we proposed that miR-30a/Slug is linked to reduced levels of fascin (*FSCN* gene); an actin-bundling protein localized to the tips of filopodia, and thus inhibits the development of the mesenchymal tumor phenotype in breast cancer. In addition, we used mouse xenotransplantation assays to demonstrate the effect of suppressing the miR-30a–directed repression of Slug on cancer cell progression. Moreover, our clinical analysis and experimental models demonstrate that the miR-30a/Slug axis is a potential therapeutic target in human breast cancer.

## RESULTS

### Decreased miR-30a expression is associated with invasiveness of breast cancer cell lines

**Figure 1:**
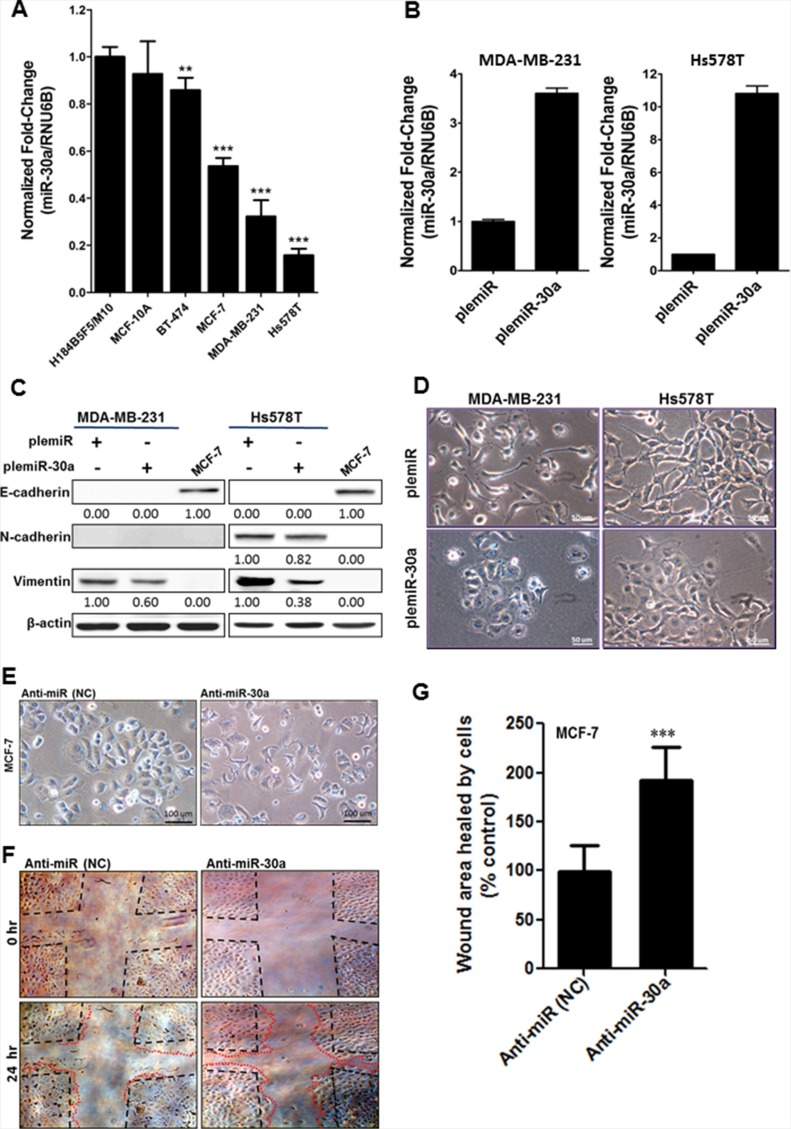
Decreased miR-30a levels in metastatic breast cancer (**A**) Comparison of miR-30a levels among normal breast epithelial cells (H184B5F5/M10 and MCF-10A) and breast cancer cell lines that are non-metastatic (BT-474 and MCF-7) or metastatic (Hs578T and MDA-MB-231). miR-30a was quantified by TaqMan real-time PCR, and the relative levels of miR-30a were normalized to *RNU6B*. (**B**) Lentiviral transduction with plemiR-30a and subsequent miR-30a overexpression in breast cancer cell lines. miR-30a levels are expressed as the mean ± SD from three independent experiments. (**C**) Western blot showing protein expression of plemiR-30a in E-cadherin–deficient breast cancer cell lines in (B), with β-actin as the loading control. (**D**) Representative images of the phenotypic change from mesenchymal to cobblestone-like epithelial cells in MDA-MB-231 and Hs578T cells transfected with plemiR-30a or plemiR (negative control). Scale bar = 50 μm. (**E**) Representative phenotypic change in MCF-7 cells transfected with an inhibitor against miR-30a (anti-miR-30a) and negative control (NC) (**F**) Representative scratch/wound healing assay images for MCF-7 cells were taken at 0 and 24 hr after scarification. (**G**) Quantification of wound healing area for MCF-7 cells as in (F). Data are expressed as the mean ± SD from triplicate experiments. ***P < 0.001 compared with the control group.

We first assessed whether decreased miR-30a expression was significantly associated with breast cancer aggressiveness in different breast cancer cell lines. miR-30a levels were significantly decreased in highly aggressive Hs578T and MDA-MB-231 breast cancer cell lines as compared with a moderate decrease in non-invasive MCF-7 and BT-474 breast cancer cell lines and in non-malignant mammary epithelial cell lines H184B5F5/M10 and MCF-10A (Figure 1A). To examine a causal link between miR-30a expression and invasiveness in Hs578T and MDA-MB-231 cells, we created a lentiviral vector that is based on plemiR; the vector contained a 551-bp fragment of the pre-miR-30a sequence (plemiR-30a) and was expressed in Hs578T and MDA-MB-231 breast cancer cells. The plemiR-30a–transfected cell lines had ~4.0- to 10-fold higher miR-30a amounts compared with plemiR-transfected control cells (Figure 1B). Interestingly, although both breast cancer cell lines are intrinsically deficient in E-cadherin (Figure 1C), indicating that they had lost an epithelial cell characteristic, the morphological change from an elongated and spindle-like fibroblastic shape to a cobblestone-like epithelial phenotype was observed when miR-30a was overexpressed (Figure 1D). In contrast, MCF-7 breast cancer cells treated with inhibitor against miR-30a (anti-miR-30a) had enhanced tumor cell motility (Figure 1E–1G), which is considered a prerequisite for retaining metastatic potential. Thus, miR-30a may have a tumor-suppressive function to inhibit the development of the mesenchymal phenotype during EMT via an E-cadherin–independent mechanism in aggressive breast cancer cells.

### miR-30a targets the 3′-UTR of *Slug* mRNA

**Figure 2:**
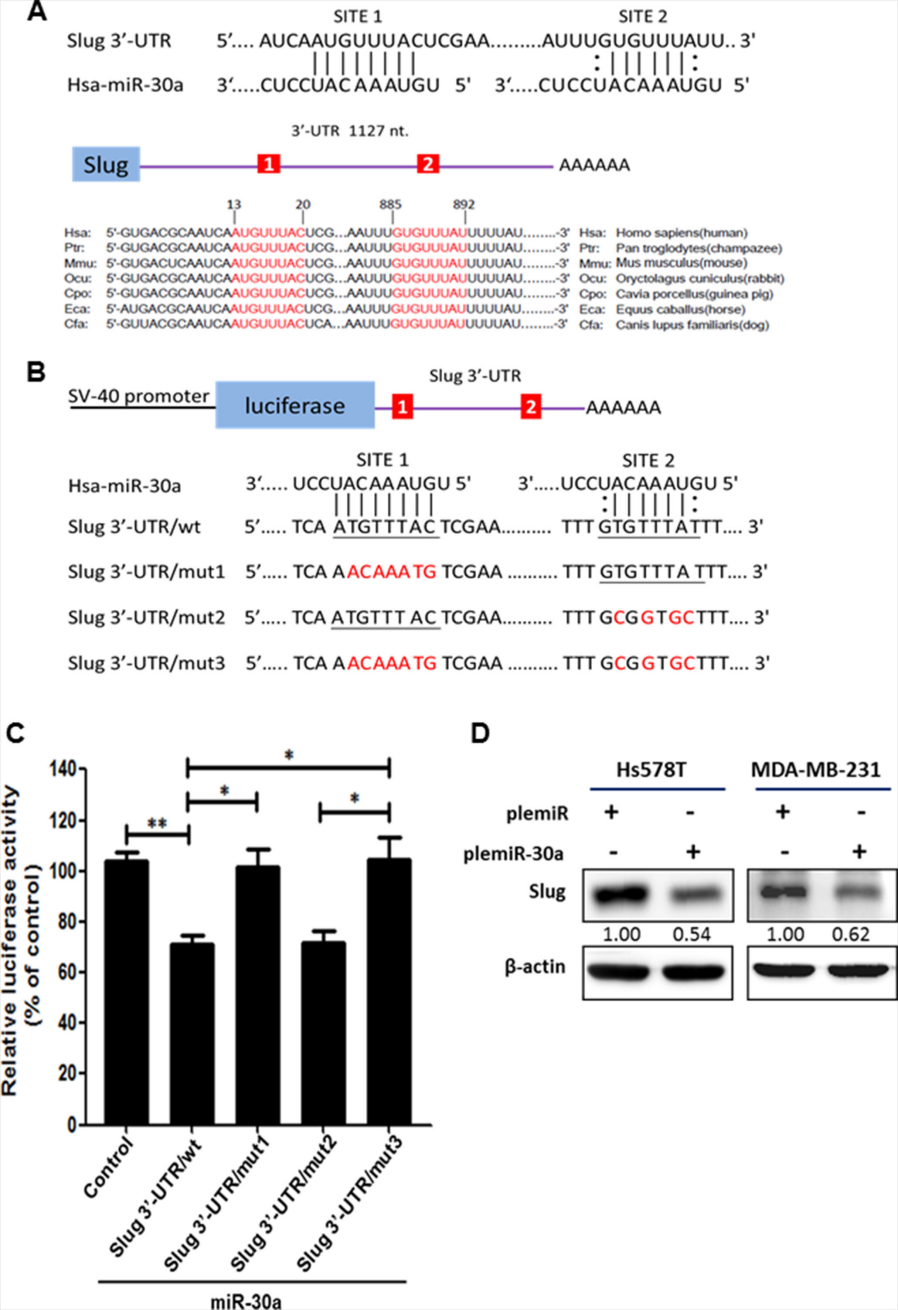
Identification of *Slug* as a downstream target for miR-30a (**A**) Predicted binding sites for miR-30a within the 3′-UTR of *Slug* mRNA. The 3′-UTR of *Slug* contains two binding regions for miR-30a (in red) across different vertebrate species. (**B**) Schematic representation of the luciferase reporter constructs showing the sequences at sites 1 and 2 of the three mutants (shown in red) with a mismatch of the miR-30a complementary sequence at site 1 (Slug 3′-UTR/mut1), site 2 (Slug 3′-UTR/mut2), or both sites (Slug 3′-UTR/mut3). The wild-type miR-30a–binding sequences are underlined. (**C**) Luciferase activity was evaluated in Hs578T cells expressing the constructs shown in (B). Firefly luciferase activity was normalized to Renilla luciferase activity and compared with the expression in cells transfected with the pcDNA3 empty vector (control). Data are presented as the mean ± SD from three independent experiments. *P < 0.05, **P < 0.01. (**D**) Western blot showing Slug expression in breast cancer cell lines transfected with plemiR-30a. β-actin was used as the loading control.

Our initial *in silico* analysis using computational prediction algorithm software, including miRanda (http://www.microrna.org/microrna/home.do), miRWalk (http://www.umm.uni-heidelberg.de/apps/zmf/mirwalk/), and TargetScan (http://targetscan.org/) predicted that *Slug* mRNA may be a target of miR-30a, and *Slug* contains two evolutionarily conserved domains in its 3′-UTR that have complementarity with human miR-30a (Figure 2A). A dual-luciferase reporter assay showed that overexpression of miR-30a reduced the activity of the luciferase gene fused to the full-length *Slug* 3′-UTR (pGL4.13/*Slug* 3′-UTR/wt) by > 30% as compared with the Hs578T-pcDNA3 cells (control group) (*P* < 0.01) (Figure 2B–2C). In addition, a significant reduction in luciferase activity was observed in the presence of pre-miR-30a using the reporter construct containing the *Slug* 3′-UTR/mut2 clone (Figure 2C). This reduction in luciferase activity was reversed by the presence of a pGL4.13 reporter construct containing mutations in the *Slug* 3′-UTR that affected either site 1 (mut1) or both site 1 and site 2 (mut3). Thus, the region from 13 to 20 is the crucial site within the 3′-UTR of *Slug* that is required for miR-30a binding. Moreover, Slug protein was notably repressed by ~ 40% in plemiR-30a–transfected Hs578T and MDA-MB-231 breast cancer cells compared with cells transfected with a control construct (Figure 2D).

### miR-30a represses Slug to increase claudins in conjunction with the MET reversion

**Figure 3:**
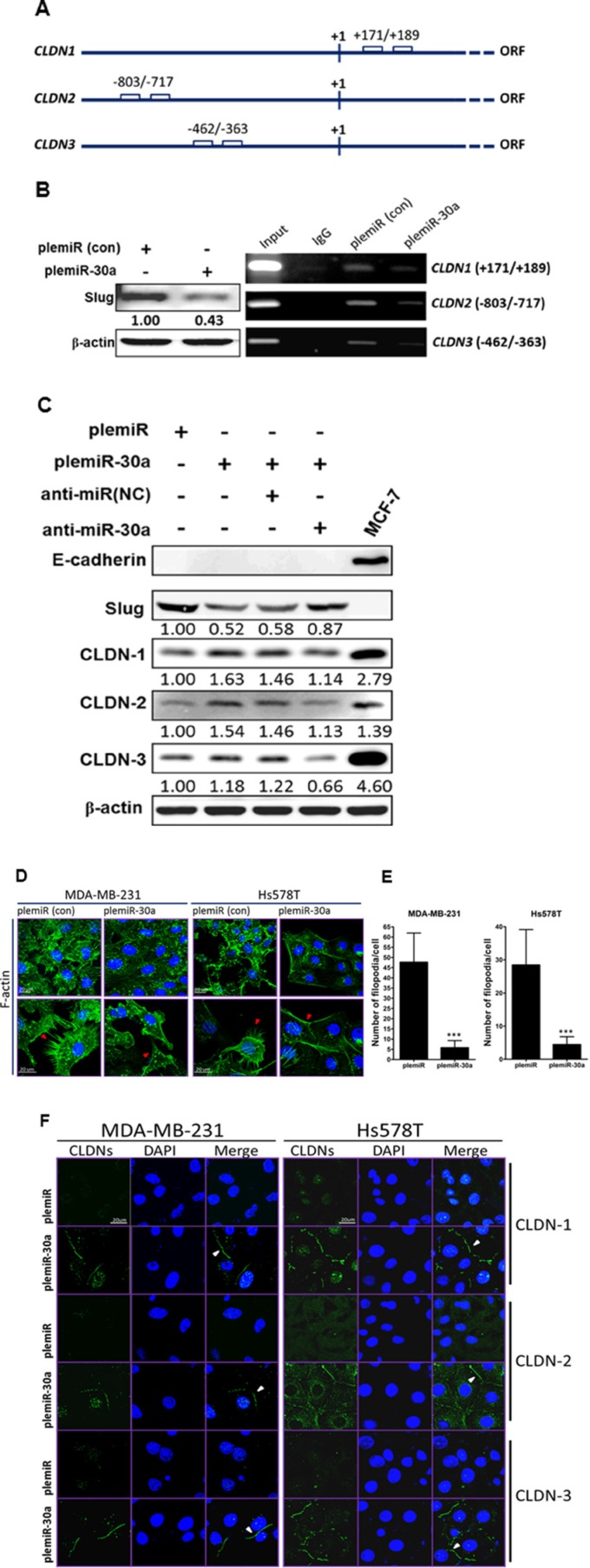
Claudin expression is enhanced by the miR-30a/Slug axis (**A**) Schematic of the E-boxes in the promoter regions of human *CLDN1*, *CLDN2*, and *CLDN3*. The starting point (+1) indicates the transcription initiation in the open reading frame (ORF) of the gene. (**B**) Western blotting (anti-Slug) of MDA-MB-231 breast cancer cell lysates after lentiviral transduction of miR-30a (left panel). PCR analysis of the genes encoding CLDN-1, -2, and -3 after ChIP in the presence of anti-Slug or anti-IgG from control MDA-MB-231/plemiR cells (con) and MDA-MB-231/plemiR-30a cells (right panel). (**C**) Western blotting revealed that Slug and fascin expression in miR-30a–overexpressing cells following miR-30a knockdown (anti-miR-30a) was inversely correlated with claudin expression. NC, negative control. (**D**) Microfilaments in MDA-MB-231 and Hs578T cells expressing plemiR-30a or the control construct plemiR were detected with Alexa Fluor 488–conjugated phalloidin (green) as indicated by red arrows. (**E**) The number of filopodial tips per cell as averaged from 50 cells per condition was calculated. The data represent the mean ± SD from three independent experiments. ***P < 0.001. (**F**) Expression of CLDN-1, -2, or -3 (green) was distributed around the cell boundary (white arrowheads) in breast cancer cell clones stably expressing miR-30a, but not in those expressing plemiR. Nuclei were counterstained in (**D**) and (**F**) with DAPI (blue). Scale bar = 20 μm.

As we demonstrated, miR-30a induction led to a diminution of the elongated and spindle-like fibroblastic phenotype in Hs578T and MDA-MB-231 invasive breast cancer cell lines (Figure 1D), in which E-cadherin is intrinsically deficient (ref. [11] and Figure 1C). We speculated that mesenchymal tumor cells may regain the cobblestone-like epithelial phenotype through activation of other Slug-regulated cellular transmembrane proteins for MET transversion, i.e., those other than E-cadherin. Because (a) claudin-based tight junction proteins are crucial for the barrier function of epithelial cell sheets in mammals [15] and (b) the E-box motif (CANNTG) in the human *CLDN1* promoter region (Figure 3A) is bound by Snail family members and Slug overexpression decreases *CLDN1* mRNA and protein [16], we thus examined whether the repression of Slug through miR-30a overexpression could increase CLDN-1 expression. Based on chromatin immunoprecipitation (ChIP) with an antibody against Slug, capture of the *CLDN1* fragment (Figure 3B) was reduced in MDA-MB-231 cells stably expressing miR-30a as compared with cells containing the plemiR empty vector. In addition, the promoter regions of *CLDN2* (nt −803 to −717) and *CLDN3* (nt −462 to −363) share the E-box motifs for Slug binding (Figure 3A), and indeed the ChIP assay showed a similar decrease in the *CLDN2*- and *CLDN3*-captured fragments in cells overexpressing miR-30a (Figure 3B). At the protein level, we also found that the levels of CLDN-1, -2, and -3 were higher in MDA-MB-231 cells transfected with plemiR-30a compared with controls (Figure 3C). In parallel, Slug expression was restored in conjunction with decreased levels of the CLDNs in plemiR-30a–transduced MD-MBA-231 cells upon transfection with an inhibitor against miR-30a (anti-miR-30a) compared with expression in the negative control (anti-miR(NC)) (Figure 3C).

Invasive cancer cells typically exhibit increased F-actin polymerization during EMT [17]. We thus examined the effect of miR-30a overexpression on the blockage of F-actin polymerization in invasive breast cancer. Labeling of MDA-MB-231 and Hs578T cells with Alexa Fluor 488–conjugated phalloidin revealed substantial disorganization of microfilaments in cells overexpressing miR-30a (Figure 3D); notably, fewer filopodia per cell were counted in miR-30a–expressing cells compared with expression of the vector alone (Figure 3E). In accordance with this phenotypic change, analysis of fluorescence images also revealed strong staining for claudins along the cell boundaries of both MDA-MB-231 and Hs578T cells stably expressing miR-30a (Figure 3F).

### miR-30a represses Slug to inhibit invasiveness of breast cancer

**Figure 4:**
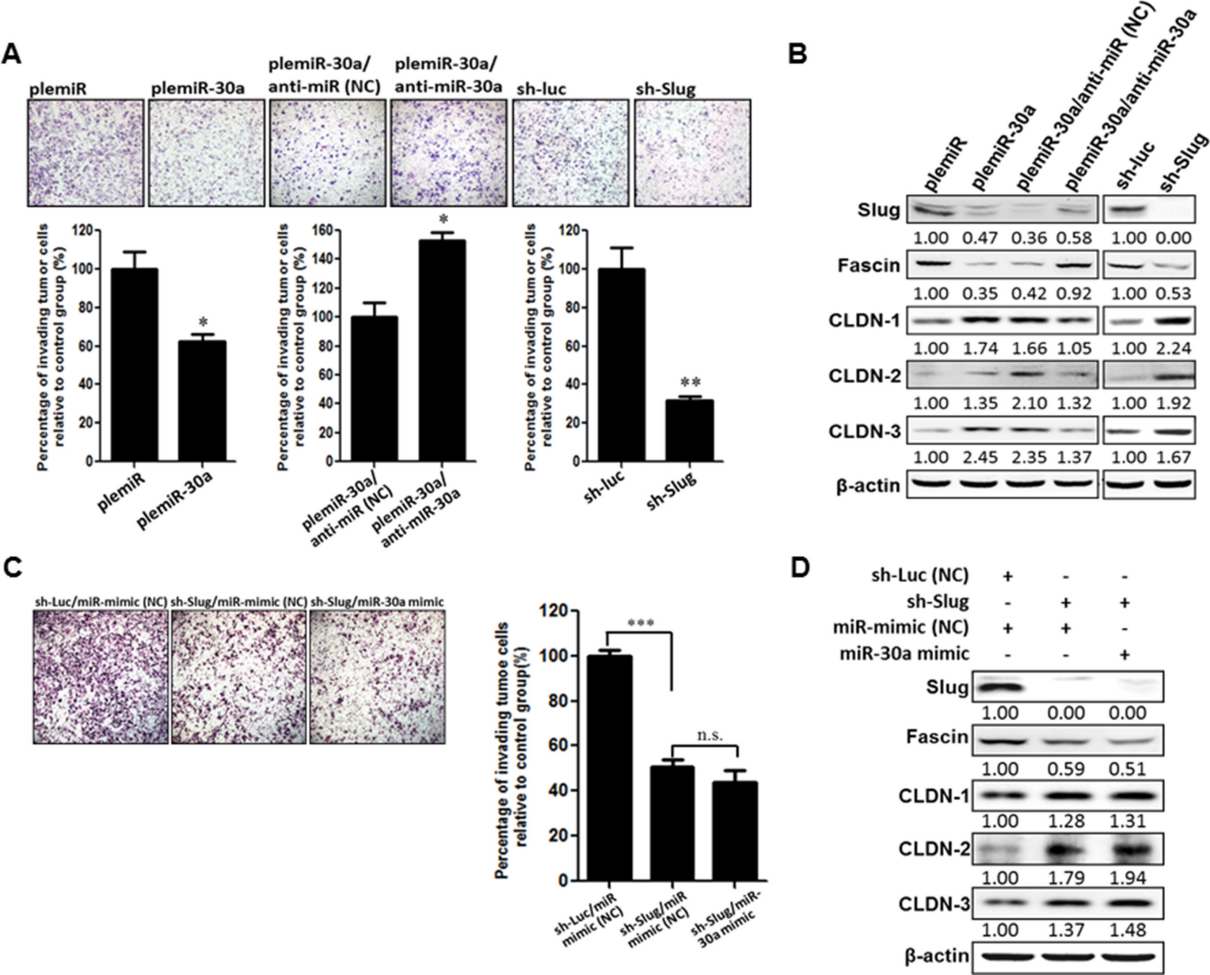
miR-30a decreases the invasiveness of breast cancer cells (**A**) MDA-MB-231 cells were transfected with plemiR or plemiR-30 and then treated with miR-30a inhibitor (anti-miR-30a) or underwent Slug knockdown with a Slug-specific short hairpin RNA (sh-Slug) or sh-Luc, a control shRNA. The cells were plated in modified Boyden chambers with polycarbonate membranes containing Matrigel and cultured for 12 h. Cells were then fixed, stained with Giemsa solution, and photographed (× 200). Upper panel: Cells that invaded through the pores onto the lower side of the filter. Lower panel: The invading cells were counted in eight randomly chosen microscope fields. Data are shown as the mean ± SD from three independent experiments. *P < 0.05, **P < 0.01. (**B**) Reduced Slug and fascin expression, but increased claudin levels, as determined by western blot analysis, in MDA-MB-231 cells expressing plemiR-30a or shRNA-Slug, as compared with controls plemiR or shRNA-Luc, respectively. In addition, Slug and fascin protein levels were restored in plemiR-30a–expressing/MDA-MB-231 cells transfected with anti-miR-30a. β-actin was the loading control. (**C**) Knockdown of Slug notably decreases the invasion of MDA-MB-231 cells, whereas overexpression of miR-30a fails to abrogate the reduced frequency of invasion for sh-Slug/MDA-MB-231 cells. (**D**) The expression of Slug, fascin, and claudins was also analyzed by western blotting. β-actin was the loading control.

Overexpression of Slug causes EMT, which leads to tumor aggressiveness by upregulation of the mesenchymal markers vimentin and fascin and down-regulation of epithelial markers E-cadherin, occludins, and claudins [18, 19]. We therefore examined the suppressive function of miR-30a in breast cancer progression in conjunction with characteristic changes in EMT markers. As expected, the number of invading cells was reduced in MD-MBA-231 cells lentivirally transduced with miR-30a by ~ 40% compared with control transduced cells (Figure 4A). Slug is required for fascin transcription and translation [18]. Thus, the reduction of Slug and fascin protein levels in the miR-30a–transduced breast cancer cells was abrogated upon transfection with the miR-30a inhibitor (anti-miR-30a), which resulted in enhanced tumor cell invasion as compared with control oligonucleotide–transfected cells (Figure 4A–4B). In parallel, knockdown of *Slug* expression in MDA-MB-231 cells increased claudin expression to inhibit cancer cell invasion (Figure 4B). Notably, this inhibition of invasion by sh-Slug treatment did not change in the presence of miR-30a overexpression (or miR control) (Figure 4C), and no changes in fascin or claudin expression were seen in sh-Slug cells overexpressing miR-30a (miR-30a-mimic) (Figure 4D). This suggests that miR-30a cannot inhibit cancer cell invasion in the absence of Slug.

### miR-30a overexpression inhibits lung colonization and tumor growth in xenograft transplantation models of human breast cancer

**Figure 5:**
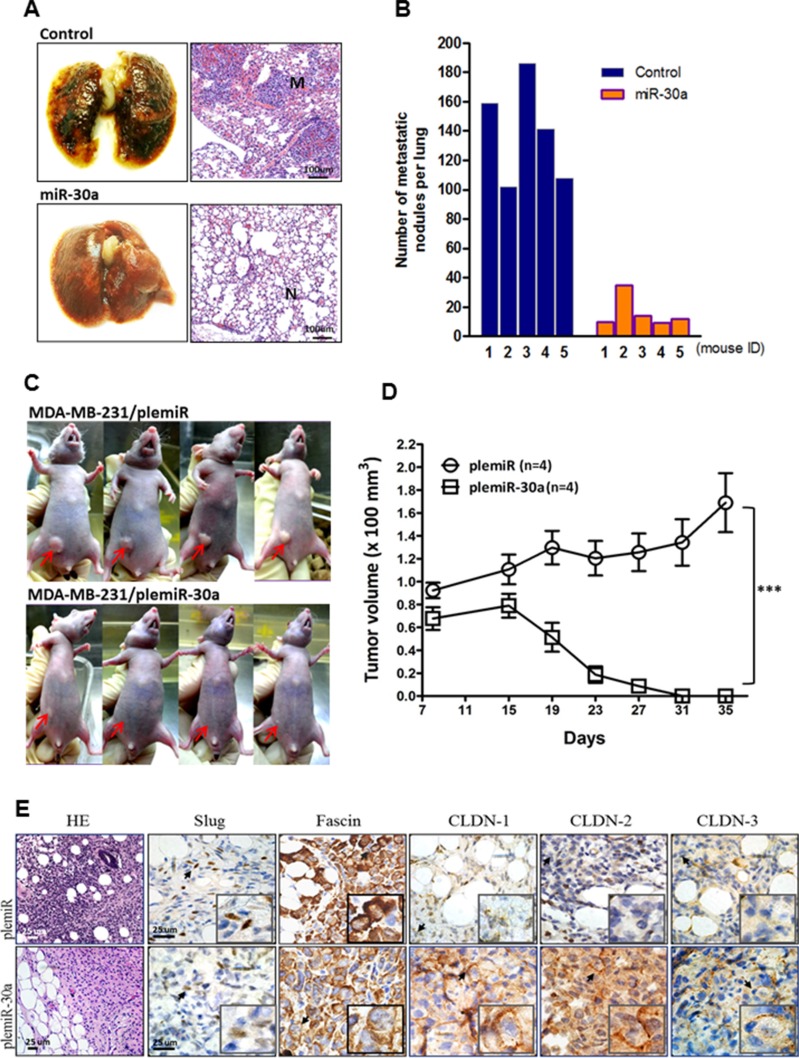
Ectopic expression of miR-30a inhibits tumor growth and metastatic lung colonization of breast cancer xenografts (**A**) Representative lungs and HE staining of metastatic tumor (M) and normal (N) lung tissues from mice 5 weeks after tail vein injection of MDA-MB-231 cells overexpressing miR-30a or plemiR vector (control). (**B**) Number of metastatic nodules in lungs of mice (*n =* 5 per group) as in (A). (**C**) Reduced tumor volumes in fat pads of nude mice injected with MDA-MB-231 cells stably overexpressing miR-30a or the control plemiR. The red arrows indicate tumors. (**D**) Xenograft tumor volumes from mice as in (C). Data represent the mean ± SD. ***P < 0.001. (**E**) Photographs of representative mice at 5 weeks post-xenotransplantation. Tumors were excised and sectioned and are shown with HE staining and specific staining for expression of Slug, fascin, and claudins (CLDN-1,–2, and–3). Scale bar = 25 μm.

Given these findings *in vitro*, we next evaluated the *in vivo* effects of miR-30a on orthotopic tumor metastasis and outgrowth with an experiment in nude mice. We established a xenograft model of human breast cancer metastasis by injection of MDA-MB-231 cells transfected with empty vector (control) or the miR-30a overexpression vector into the tail vein of 6-week-old mice. After 5 weeks, mice were sacrificed and their lungs dissected to evaluate tissue morphology by hematoxylin and eosin (HE) staining (Figure 5A). Tumors with high expression of miR-30a formed only a few pulmonary metastatic nodules on average (16.0 ± 10.8) in all mice analyzed and significantly fewer than the number of nodules formed in the control group (139.2 ± 35.2, *P =* 0.0028) (Figure 5B). We also injected breast tumor cells into the mammary fat pads of mice to study the effect of miR-30a on inhibition of tumor outgrowth. After 4 weeks, the subsequent tumors in the mice injected with MDA-MB-231 cells that overexpressed miR-30a were significantly smaller than those in the control group (*P* < 0.001) (Figure 5C–5D). Breast tumor tissues from mice were resected and then subjected to immunohistochemical staining. The resultant positive staining for CLDN-1, -2, and -3 that was associated with decreased expression of Slug and fascin differentiated the orthotopic tumor tissues with miR-30a overexpression from those carrying vector alone (Figure 5E). This supported a suppressive function for miR-30 in breast cancer invasiveness and metastasis *in vivo*.

### The miR-30a^low^/*CLDN*^low^/*FSCN*^high^ genotype in association with breast cancer progression

**Figure 6:**
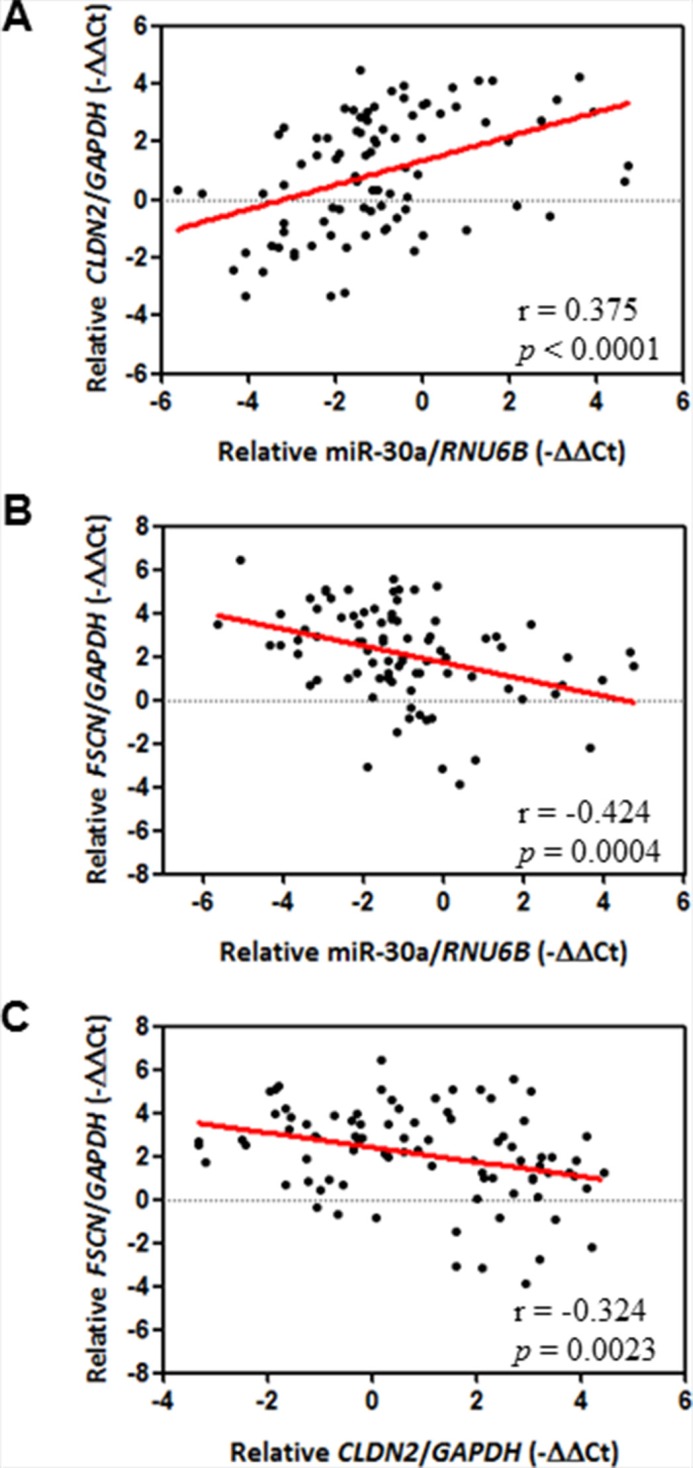
Correlation between miR-30a and EMT markers The Pearson correlation coefficient was used to analyze mRNA expression data from 86 patients with breast cancer. (**A**) Positive correlation between miR-30a and epithelial marker *CLDN2*. (**B**) Inverse correlation between miR-30a and mesenchymal marker *FSCN* (**C**) Inverse correlation between *CLDN2* and *FSCN*. The relative expression of each mRNA was calculated using the comparative CT method.

Our results at the molecular and cellular levels were thus consistent with those from an animal model, all of which indicated that increased expression of CLDN-1, CLDN-2, and CLDN-3 and decreased expression of fascin are controlled by the miR-30a/Slug axis. We therefore determined whether there is an association between miR-30a/claudin/fascin and clinicopathological significance of breast cancer. We used laser capture microdissection to isolate tumor cells from specimens to avoid contamination with normal tissue and then measured the expression of individual genes by quantitative real-time PCR. In our breast cancer cohort (*n =* 86) (Table 1), *CLDN2* mRNA transcripts were significantly and positively associated with miR-30a levels in cancerous tissues (Pearson correlation coefficient, 0.375; *P =* 0.0004) (Figure 6A). In contrast, *FSCN* mRNA had an opposite correlation with miR-30a (Pearson correlation coefficient, −0.424; *P* < 0.0001) (Figure 6B). In addition, a high level of *CLDN2* mRNA was correlated with a low level of *FSCN* mRNA (Pearson correlation coefficient, −0.324; *P* = 0.0023) (Figure 6C).

**Table 1:** Tumor clinicopathological features of female patients with breast cancer

Clinicopathological feature	*n* (%)
Age (mean ± SD and range)	50.3 ± 12.6 (23–87 yr)
Tumor size (mm)	
≤ 20	42 (48.8)
> 20	44 (51.2)
Histologic grade	
I	13 (15.1)
II	40 (46.5)
III	33 (38.4)
AJCC stage^a^	
I	27 (31.4)
IIa	27 (31.4)
IIb	14 (16.3)
III	15 (17.4)
IV	3 (3.5)
Tumor extension in metastatic lymph node	
LNM-negative (N_0_)^b^	41 (47.7)
LNM-positive (N_1_/N_2_)	45 (52.3)

a Tumor classification was based on the sixth edition of the AJCC Cancer Staging Manual [40].

b LNM, lymph node metastasis.

To assess the prognostic prediction of the interaction between miR-30a and *FSCN* or between miR-30a and *CLDN2* in breast cancer, we defined expression status as “high” (≥ 4.90-fold increase in *FSCN* mRNA compared with the median) or “low” (< 2.40-fold decrease in *CLDN2* mRNA) by comparing expression in cancer cells and adjacent non-cancerous cells. With miR-30a^high^/*FSCN*^low^ and miR-30a^high^/*CLDN2*^high^ as the reference, there was a greater proportion of the miR-30a^low^/*FSCN*^high^ and miR-30a^low^/*CLDN2*^low^ genotype, respectively, in cancer patients with a large tumor size, advanced tumor stage, or lymph node involvement (*P* for trend < 0.05) at the time of diagnosis (Table 2).

**Table 2:** Effect of interaction between miR-30a and *FSCN* or between miR-30a and *CLDN2* transcripts on poor prognosis in breast cancer

			Clinicopathological characteristic						
Genotype variant ^a^	pT (*n*, %)			LNM (*n*, %)^b^			Stage (*n*, %)^c^		
	> 20 mm	≦ 20 mm	OR (95%CI)^d^	LNM (+)	LNM (−)	OR (95%CI)	Late stage	Early stage	OR (95%CI)
miR-30a^high^/Fascin^low^	10 (40.0)	15 (60.0)	1.00 (Ref)	10 (40.0)	15 (60.0)	1.00 (Ref)	4 (16.0)	21 (84.0)	1.00 (Ref)
miR-30a^high^/Fascin^high^	5 (45.5)	6 (54.5)	1.25 (0.32−5.23)	5 (45.5)	6 (54.5)	1.25 (0.32−5.23)	4 (36.4)	7 (63.6)	2.99 (0.59−15.29)
miR-30a^low^/Fascin^low^	7 (41.2)	10 (58.8)	1.06 (0.35−3.68)	7 (41.2)	10 (58.8)	1.05 (0.35−3.68)	6 (35.3)	11 64.7)	2.86 (0.67−12.34)
miR-30a^low^/Fascin^high^	22 (66.7)	11 (33.3)	3.00 (1.02−8.83)*	23 (69.7)	10 (30.3)	3.45 (1.16−10.28)*	18 (54.5)	15 (45.5)	6.30 (1.76−22.42)**
			*P* for trend = 0.054			*P* for trend = 0.031			*P* for trend = 0.005
miR-30a^high^/CLDN2^high^	7 (31.8)	15 (68.2)	1.00 (Ref)	7 (31.8)	15 (68.2)	1.00 (Ref)	3 (13.6)	19 (86.4)	1.00 (Ref)
miR-30a^high^/CLDN2^low^	8 (57.1)	6 (42.9)	2.85 (0.71−11.43)	8 (57.1)	6 (42.9)	2.85 (0.71−11.43)	5 (35.7)	9 (64.3)	3.52 (0.70−18.07)
miR-30a^low^/CLDN2^high^	11 (52.4)	10 (47.6)	2.36 (0.68−8.15)	9 (42.9)	12 (57.1)	1.61 (0.46−5.58)	7 (33.3)	14 (66.7)	3.17 (0.72−14.46)
miR-30a^low^/CLDN2^low^	18 (62.1)	11 (37.9)	3.51 (1.09−11.29)*	21 (72.4)	8 (27.6)	5.62 (1.67−18.89)**	17 (58.6)	12 (41.4)	8.97 (2.16−37.28)**
			*P* for trend = 0.051			*P* for trend = 0.012			*P* for trend = 0.002

a Expression status of the individual mRNAs was defined by comparing target mRNA expression in tumor cells and adjacent non-tumor cells captured from the primary tumor site of the same patient and was calculated by the comparative CT method. Determination of the “high” or “low” expression of the individual miRNA and mRNA targets was as described in the Results.

b Lymph node status was classified as N_0_ (LNM-negative) and N_1_, and N_2_ (LNM-positive).

c Tumors were categorized as early stage (stage I and IIa) and late stage (stage IIb/III/IV) based on the sixth edition of the AJCC Cancer Staging Manual [40].

d OR (95%CI), odds ratio and 95% confidence interval. Ref, reference group. *P < 0.05 and **P < 0.01.

We next addressed the question regarding joint effects of miR-30a^low^/*CLDN2*^low^/*FSCN*^high^ on prognostic assessment of breast cancer. A joint effect of higher risk associated with poor clinicopathological features of breast cancer was observed in patients who more closely adhered to the miR-30a^low^/*CLDN2*^low^/*FSCN*^high^ genotype (Table 3). We analyzed the statistical significance with the trend test for one additional risk genotype in individuals with a large tumor size, lymph node metastasis, or advanced tumor stage as measured by the β estimates from the regression model (*P* for trend < 0.05) (Table 3).

**Table 3:** Additive effect of the tumors with greater numbers of deregulated mRNAs (miR-30a, *CLDN2*, and *FSCN*) on clinicopathological characteristics in breast cancer

Number of deregulated mRNAs relative to miR-30a^low^*/CLDN2*^low^/*FSCN*^high^ ^a^	Clinicopathological characteristic					
	pT (> 20 mm)		LNM-positive		Tumor stage (III/IV)	
	*n* (%)	OR (95%CI)^b^	*n* (%)	OR (95%CI)	*n*(%)	OR (95%CI)
0	7 (38.9)	1.00 (Ref)	6 (33.3)	1.00 (Ref)	3 (16.7)	1.00 (Ref)
1	8 (36.4)	0.90 (0.25−3.24)	8 (36.4)	1.14 (0.31−4.23)	4 (18.2)	1.12 (0.21−5.76)
2	13 (56.5)	2.04 (0.58−7.17)	14 (60.9)	3.11 (0.86−11.29)	11 (47.8)	4.58 (1.04−20.24)*
3	16 (69.6)	3.59 (0.98−13.16)	17 (73.9)	5.67 (1.47−21.89)*	14 (60.9)	7.78 (1.74−34.72)**
Additive model of deregulated miRNAs		1.62 (1.08−2.43) *P* for trend = 0.021		1.89 (1.23−2.89) *P* for trend = 0.003		2.21 (1.38−3.54) *P* for trend = 0.001

a Cutoffs of miR-30a^low^, *CLDN2*^low^, and *FSCN*^high^ genotypes were as described in the Results.

b ORs and 95%CIs were estimated in a logistic regression model, in which a group of dummy variables was used to represent different groups of patients showing different numbers of risk genotypes. *P < 0.05; **P < 0.01.

**Figure 7:**
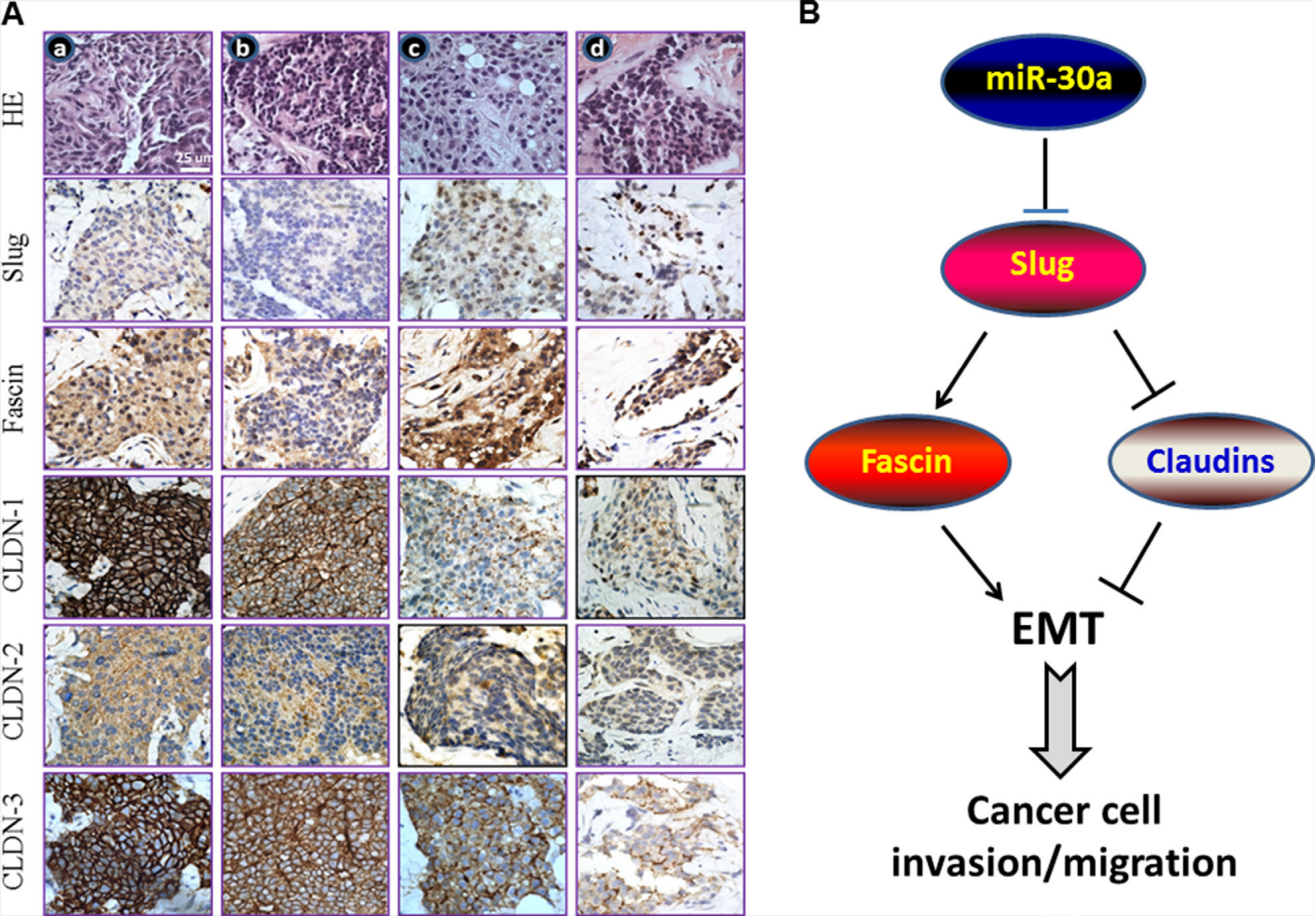
miR-30a inhibits EMT by binding to Slug (**A**) The cellular phenotype of miR-30a^low^/Slug^high^/Fascin^high^/Claudin^low^ correlates with poor clinicopathological features in breast cancer. Immunostaining results for paraffin-embedded breast cancer tissue samples of LNM-negative and early-stage tumors (stage I and IIa; columns a and b) and LNM-positive and advanced-stage tumors (stages III and IV; columns c and d). In addition, the T/N ratio for miR-30a was < 0.50 in columns c and d and was ≥ 0.50 in columns a and b. Clinicopathological features of the tumors were determined according to the sixth edition of the AJCC Cancer Staging Manual [40]. (**B**) The tumor-suppressor role of miR-30a relative to its inhibition of EMT. The targeting of *Slug* mRNA by miR-30 results in downregulation of fascin and upregulation of the tight junction proteins CLDN-1, CLDN-2, and CLDN-3, which downregulates EMT and, ultimately, reduces the rate of breast cancer progression.

More specifically, the differences in the expression of the proteins encoded by these genes were validated by immunohistochemistry in breast cancer tissue specimens, and the results were stratified based on the miR-30a level (Figure 7A). In the immunohistochemical analysis (*n* = 10), tissues that expressed high levels of miR-30a (tumor-to-normal (T/N) ratio ≥ 0.50-fold as defined in [10]), had intense positive staining for the three claudin proteins, and had reduced or undetectable levels of Slug and fascin were from well-differentiated, lymph node metastasis (LNM)-negative, and non-invasive tumors. In contrast, the advanced breast tumor tissues (late stage and LNM-positive) with decreased (T/N ratio < 0.50-fold) or undetecTable miR-30a (T/N ratio < 0.10-fold) had low-intensity staining for claudins but strong intensity for Slug and fascin (Supplementary Table S1).

## DISCUSSION

miR-30a inhibits EMT in different types of cancer, including gastric, liver, and lung cancer [12, 20, 21]. Here we propose a mechanism for this effect by which miR-30a counteracts the aggressiveness and metastasis of cancer cells by increasing tight junction molecules—CLDN-1, CLDN-2, and CLDN-3—via targeting the 3′-UTR of *Slug* mRNA. This increased expression of claudins can support tight junction function, which may regulate EMT and eventually prevent breast cancer progression (Figure 7B). Together, these findings may help to develop a candidate miRNA biomarker that could lead to prognostic assessment for breast cancer patients.

The *in silico* prediction, luciferase reporter assay, and western blotting all indicated that *Slug* mRNA is a direct target of miR-30a. According to data sorting of the mRNA sequences bound to miRNAs, miR-30 family members (miR-30a, -30b, -30c, -30d, and -30e) share the same seed sequence (Supplementary Figure S1), suggesting that other miR-30 family members may also suppress Snail or Slug. Indeed, decreased luciferase activity in a reporter assay showed that the 3′-UTR of *Snail* mRNA is a direct target of miR-30a in breast cancer cells (Supplementary Figure S2), and this is consistent with a previous report that miR-30a overexpression downregulates Snail and thereby inhibits invasion and metastasis of lung carcinomas [12]. Likewise, miR-30c is significantly downregulated in human renal cell carcinomas, in which miR-30c overexpression may suppress EMT by binding *Slug* mRNA to increase E-cadherin production [22]. Additional studies are needed to determine whether defects in miR-30 family members act independently or jointly to drive the progression of breast cancer.

Slug can promote the formation of filopodia-like bundles via transcriptional activation of *FSCN* [18, 23] to sustain filopodial tips during the metastasis and invasion of cancer cells [24, 25]. The miR-30a/Slug axis inhibited filopodial assembly during EMT in breast cancer cells, which resulted in reduced levels of mesenchymal proteins, e.g., vimentin and fascin. miR-30 family members, including miR-30a, are downregulated in estrogen receptor–negative and progesterone receptor–negative breast tumors, suggesting that these hormones are involved in *de novo* synthesis of miR-30 family members [26, 27]. We are currently mapping the specific region that harbors the hormone-response element(s) in the miR-30 promoter and will identify the hormonal mechanism that regulates miR-30 expression, which could help determine the clinical benefit of endocrine therapy in individuals with hormone receptor–positive breast cancer.

Additional miR-30a–regulated targets have been identified and their functions defined, including involvement in tumor cell autophagy [28], mediating cis-platinum chemosensitivity [29], suppressing metastatic colorectal cancer by inactivating the Akt/mTOR pathway [30], and inhibiting breast cancer metastasis by decreasing metadherin [31]. Our present miRNA study demonstrates that Slug, as well as vimentin [10], is a miR-30a target that is particularly important in breast cancer progression. In addition, clinical observations, including those of our breast cancer cohort [10, 31], showed that miR-30a reduction is associated with lymph node and lung metastases in patients with breast cancer. Importantly, miR-30a inhibits the attachment-independent growth of breast tumor–initiating cells identified in a subset of tumors with unlimited self-renewal and differentiation heterogeneity [32]. The exclusively tumor-suppressive effect of miR-30a in the regulation of multiple important tumorigenic genes/pathways involved in cancer cell heterogeneity may drive the development and evaluation of miR-30a as a therapeutic for breast cancer.

## CONCLUSIONS

The tumor-suppressive function of miR-30a reverses EMT in breast cancer by directly targeting the 3′-UTR of *Slug*. *In vitro* binding of miR-30a to *Slug* mRNA increased expression of the tight-junction proteins CLDN-1, -2, and -3 and decreased the metastatic capability of those cells owing to its effects on the reduction of F-actin polymerization. Consequently, tumor progression was suppressed in mouse xenotransplantation assays. Clinically, RNA expression profiles and immunohistochemical analyses confirmed that the miR-30a^low^/Claudin^low^/Fascin^high^ link correlated with poor prognosis for breast cancer. Thus, miR-30a may be useful as a therapeutic strategy for breast cancer treatment.

## MATERIALS AND METHODS

### Cell culture

The human mammary epithelial cell line, MCF-10A, was a kind gift of Dr. Yung-Luen Yu at the Graduate Institute of Cancer Biology, China Medical University, Taichung, Taiwan. MCF-10A cells were maintained in DMEM/F12 medium (Life Technologies, Carlsbad, CA, USA) containing 0.1 mM sodium pyruvate, 5% horse serum, 10 μg/mL insulin, 2 mM L-glutamine, 100 IU/mL penicillin, 100 μg/mL streptomycin, 20 ng/mL epidermal growth factor, 500 ng/mL hydrocortisone, and 100 ng/mL cholera toxin in a humidified 5% CO_2_ atmosphere at 37°C. The breast cancer cell lines (Hs578T, MDA-MB-231, MCF-7, and BT-474) and the non-malignant mammary epithelial cell line H184B5F5/M10 were obtained from the American Type Culture Collection (Manassas, VA, USA) and cultured in DMEM (Life Technologies) containing 0.1 mM sodium pyruvate, 10% FBS, 2 mM L-glutamine, 100 IU/mL penicillin, and 100 μg/mL streptomycin.

### Cell lysis and western blotting

A detailed procedure for western blotting is described elsewhere [10]. Primary antibodies against human Slug and vimentin (Cell Signaling Technology, Danvers, MA, USA); CLDN-1, -2, and -3 (Novus Biologicals, Littleton, CO, USA); and E-cadherin, N-cadherin, and fascin (Santa Cruz Biotechnology, Dallas, TX, USA) were used. The antibody against β-actin, which was used as the endogenous control to normalize the expression of proteins of interest, was obtained from Sigma-Aldrich (St. Louis, MO, USA). An appropriate horseradish peroxidase–conjugated secondary antibody was used, and the immunoreactive protein was visualized with an enhanced chemiluminescence assay (Western Blotting Luminol Reagent; Santa Cruz Biotechnology). The band intensities were quantified by densitometry (Digital Protein DNA Imagineware, Huntington Station, NY, USA).

### Wound healing assay

To determine the migratory behavior of MCF-7 cells, a bidirectional wound healing assay was performed [33]. Briefly, cells were grown in medium to monolayer confluence in 24-well culture plates. Cells were treated with anti-miR-30a or anti-miR-mimic (NC), and after which a sterile 10-μL tip was used to scratch the monolayer of cells to form a bi-directional wound. The plate was kept in a 37°C, 5% CO_2_ incubator overnight. Pictures of a representative field of the cell-free space were taken at 0 and 24 hr after the scratch using a microscope, and the distance of cell migration was calculated with Image Pro plus software (Media Cybernetics, Silver Spring, USA).

### Dual luciferase reporter assay

The 3′-UTR sequence of human *Slug* was cloned into pGL4.13 (Promega) to produce the recombinant vector pGL4.13/Slug 3′-UTR wt, which also contains the firefly luciferase ORF under the control of the SV40 promoter. Two miR-30a sites complementary to the *GTTTAC* sequence in the *Slug* 3′-UTR were mutated individually or in combination to remove complementarity to miR-30a using the QuikChange II XL site-directed mutagenesis kit (Stratagene, La Jolla, CA, USA) with pGL4.13/Slug 3′-UTR/wt as the template. The mutants were named Slug 3′-UTR/mut1 (single mutant), 3′-UTR/mut2 (single mutant), and 3′-UTR/mut3 (double mutant). Supplementary Table S2 lists the primer sequences with mutated nucleotides underlined; also shown are the sequences of the mismatch primers used to generate the different *Slug* 3′-UTR mutants. MDA-MB-231 cells in 24-well plates were co-transfected with 100 ng *Slug* reporter construct containing wild-type or mutated 3′-UTR and pcDNA3 (control) or pcDNA3/miR-30a.

### Establishment of breast tumor cells stably expressing miR-30a

Lentivirus carrying hsa-miR-30a (plemiR-30a) or control (plemiR) was packaged with a lentivirus expression system (Thermo Fisher Scientific) and the Trans-Lentiviral^TM^ GIPZ Packaging System (Open Biosystems, Huntsville, AL, USA). A puromycin-resistant selectable marker was used to select against non-transduced cells to amplify miR-30a from the Hs578T and MDA-MB-231 cells.

### ChIP

Chromatin from cell lines was sonicated and immunoprecipitated with rabbit polyclonal antibody against Slug or with rabbit IgG (negative control). The chemical cross-links were reversed by overnight incubation at 65°C in the presence of 8 M NaCl, followed by the addition of proteinase K (10 mg/mL) for 1 h at 45°C and RNase (10 mg/mL) for 30 min at 37°C. After extraction and precipitation, DNA was dissolved in 30 mL of ddH_2_O. Primers (Supplementary Table S2) were used to amplify specific sections of the 150-bp *CLDN1*, *CLDN2*, and *CLDN3* promoter regions that contain the predicted Slug binding sites. The PCR products were analyzed by 2% agarose gels and visualized with ethidium bromide staining.

### Invasion assay

The cells were trypsinized and collected from dishes via brief centrifugation. Samples consisting of 5 × 10^4^ cells were seeded into 48-well modified Boyden chambers (Neuro Probe, Cabin John, MD, USA) with 8-μm pore size polycarbonate membrane filters with Matrigel for 12 h, and invading cells that were attached to the lower surface of the membrane were fixed with methanol and stained with Giemsa solution (Sigma-Aldrich Co., St Louis, MO, USA). Invading cells were quantified by counting five random high-power fields using an Olympus Ckx41 light microscope (Tokyo, Japan.).

### Confocal microscopy

Cells were prepared for confocal laser scanning microscopy as described [34]. Cells were incubated separately with mouse anti-CLDN-1 (Invitrogen) or rabbit anti-CLDN-2 or anti-CLDN-3 (Novus Biologicals). For F-actin staining, cells were incubated with Alexa Fluor 488–conjugated phalloidin (Invitrogen) diluted in a blocking solution (1:40) for 20 min. Nuclei were stained with 4′,6′-diamidino-2-phenylindole (DAPI). The samples were examined under a confocal laser scanning microscope (Zeiss LSM 510 META, Jena, Germany) equipped with a UV laser (351/364 nm), an argon laser (457/488/514 nm), and a helium/neon laser (543 nm/633 nm).

### Mouse xenotransplantation assay

All mice were housed in the animal facility at the Chung Shan Medical University Experimental Animal Center, Taichung, Taiwan. Approval was obtained from the Institutional Animal Care and Use Committee of Chung Shan Medical University for the use of animals, and all experiments were performed in accordance with the guidelines for animal care of that committee. For the orthotopic implantation model, 6- to 8-week-old female BALB/c nude mice were used. The mice were injected through the mammary fat pad with MDA-MB-231 breast tumor cells (1 × 10^6^) suspended in Matrigel (20% v/v) (Becton-Dickinson, Franklin Lakes, NJ, USA) in PBS. The detailed procedure for measurement of tumor volume via an external caliper is described elsewhere [35]. In this way, primary tumors were measured on the days indicated in Figure 5, and tumor volume was calculated using the formula 1/2 (length × width^2^). In addition, the breast cancer lung metastasis model was established by tail-vein injection of MDA-MB-231 cells (5 × 10^5^ cells in 0.1 mL of PBS). After 5 weeks, mice were sacrificed by CO_2_ asphyxiation. The number of metastatic lung tumors was confirmed with HE staining under a dissecting microscope.

### Laser-capture microdissection and quantitative real-time PCR

All frozen tissue specimens were confirmed to be primary breast invasive ductal carcinoma based on their pathological features, and all the participants provided their written informed consent to participate in this study. Considerations regarding methodological issues in the present study, including research design, sampling scheme, and consent procedure, were approved by the Ethics Committee of the Institutional Review Board at the Chung Shan Medical University Hospital, Taichung, Taiwan. A detailed procedure for RNA isolation from cells collected by laser-capture microdissection is described elsewhere [10, 36]. RNAs were extracted from laser-capture microdissection–>collected samples of the tumor and the adjacent non-tumor breast tissue of each patient using the mirVana miRNA isolation kit (Ambion Inc., Austin, TX), and the RNA concentration in each sample was quantified on a NanoDrop 1000 spectrophotometer (NanoDrop Technologies, Waltham, MA). The single-tube TaqMan miRNA assay (Applied Biosystems, Foster City, CA, USA) was used to detect and quantify mRNA expression on an Applied Biosystems instrument. Appropriate probes and primer sets were used to detect expression of genes encoding hsa-miR-30a (AB assay ID: 000417), *FSCN* (Hs00362704_m1), and *CLDN2* (Hs01549234_m1) according to the procedure described by Applied Biosystems. Results were normalized against *GAPDH* (for *FSCN* and *CLDN2*) and *RNU6B* (for miR-30a). The relative expression of each mRNA, i.e., the Ct value, was calculated for the microdissected cells from the tumor and paired non-tumor tissues using the comparative CT method [10].

### Immunohistochemistry

Detailed procedures for the immunohistochemical assays and scoring system are described elsewhere [36, 37]. Briefly, each tissue slide was reacted with monoclonal antibodies against Slug (Cell Signaling Technology); fascin (Santa Cruz Biotechnology); or CLDN-1, -2, or -3 (Novus Biologicals) in a humidified chamber for 60 min at 37°C. Afterwards, the sections were washed with PBS and treated with an appropriate biotin-conjugated secondary antibody for 30 min. The signal for each tissue section was detected using the avidin-biotin complex system and diaminobenzidine kit (Vector Laboratories, Burlingame, CA, USA). Immunohistochemical staining was quantified according to the coverage area and intensity for Slug as described [38]. The scoring system for the CLDNs and fascin is described elsewhere [39], with the following modifications: –, no staining or membrane staining in < 10% of tumor cells; +, faint, weakly perceptible positive staining of the membrane in < 10% of tumor cells; ++, weak-to-moderate complete membrane staining observed in ≥ 10% of tumor cells; and +++, strong, complete membrane staining in ≥ 10% of tumor cells.

### Statistical analysis

Data are presented as the mean ± SD. Statistical comparisons were performed using the Student's *t*-test to test any statistically significant difference in the results between the different test groups. Statistical significance of the experimental data grouped by one variable was assessed with an unpaired two-tailed Student's *t*-test or a one-way ANOVA followed by Dunnett's test. All statistical analyses were performed using SPSS version 17.0 (SPSS Inc., Chicago, IL, USA).

## SUPPLEMENTARY MATERIALS FIGURES AND TABLES


