# Supplementary material for: MicroRNA-30a increases tight junction protein expression to suppress the epithelial-mesenchymal transition and metastasis by targeting Slug in breast cancer
Source: Oncotarget. 2016 Feb 24;7(13):16462–78. doi: 10.18632/oncotarget.7656 (PMC4941328; doi:10.18632/oncotarget.7656)
Supplement: Supplementary file 1 [file oncotarget-07-16462-s001.pdf]

MicroRNA-30a increases tight junction protein expression to suppress the epithelial-mesenchymal transition and metastasis by targeting Slug in breast cancer

Supplementary Materials

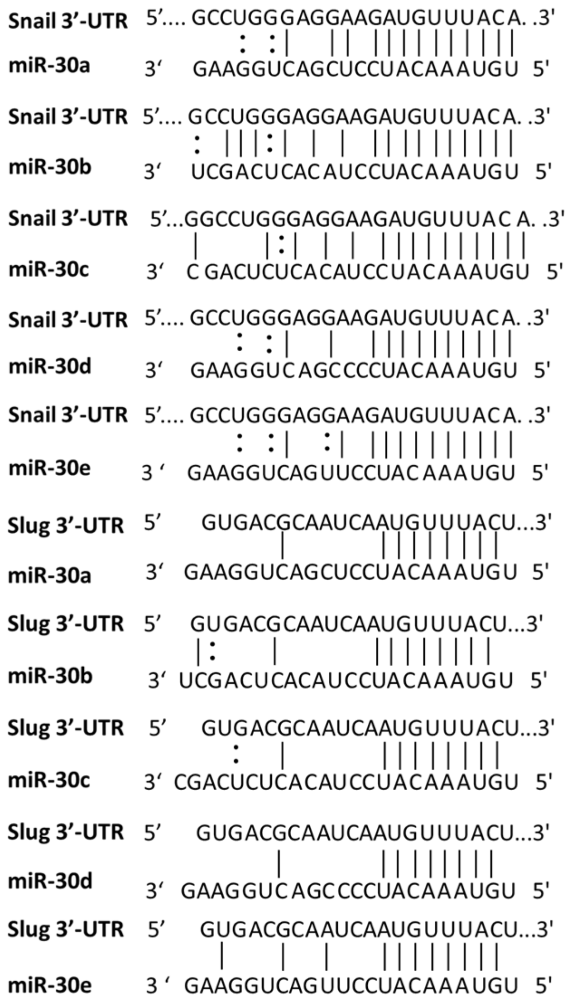

Supplementary Figure S1: Prediction of the sequences within Snail mRNAs targeted by miR-30 family members. Computational algorithm results show that miR-30 family members share the same seed sequence, which targets the 3′-UTRs of *Snail* and *Slug*.

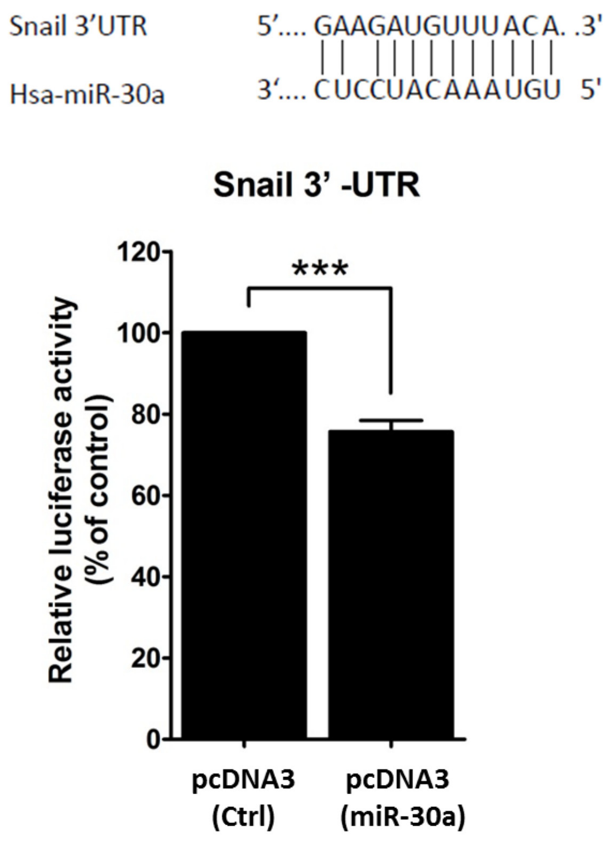

Supplementary Figure S2: *Snail* as a downstream target of miR-30a. Three algorithms, miRanda, miRWalk, and TargetScan, were used to identify putative interaction sites between *Snail* 3′-UTR and miR-30a. The consensus binding site in the *Snail* mRNA is shown. Expression of miR-30a reduced the activity of the luciferase gene fused to the *Snail* 3′-UTR by > 20%, supporting the notion that miR-30a downregulates Snail levels by targeting the 3′-UTR of the mRNA. The control (Ctrl) consisted of the *Snail* 3′-UTR construct along with pcDNA3.

Supplementary Table S1: Immunohistochemical analysis of slug, fascin, and claudins stratified by miR-30a expression levels in association with clinicopathological features of breast cancer

Cliniopathological features	miR-30a expression (T/N ratio)[a]	Slug	Fascin	CLDN-1	CLDN-2	CLDN-3
pT1, N1, Stage I	> 0.5	+/–	+	++	+++	+++
pT1, N0, Stage IIa	> 0.5	–	–	+++	++	+++
pT2, N1, Stage IIb	< 0.5	+++	++	–	+	–
pT2, N1, Stage IIb	< 0.5	+++	++	–	–	+/–
pT2, N2, Stage IIIa	< 0.5	+++	++	+	+/–	–
pT2, N2, Stage IIIa	< 0.1	+++	+++	+	–	+
pT2, N2, Stage IV	< 0.1	+++	+++	+	+/–	–
pT4, N2, Stage IIIb	< 0.1	++	++	+	–	+
pT4, N2, Stage IIIb	< 0.1	+++	+++	–	–	+/–
pT1, M1, Stage IV	< 0.1	+++	+++	–	–	–

[a]Detailed quantitation for expression level of miR-30a in tumor tissues was described elsewhere [10]. The expression status of the miR-30a was defined by comparing miR-30a expression in tumor cells and adjacent non-tumor cells captured from the primary tumor site of the patient as described accordingly. Immunohistochemical staining and scoring system for the paraffin-embedded breast cancer tissue samples from patients were performed and asessed as described in Material and method. Tumor classification was referred to sixth edition of the AJCC Cancer Staging Manual [40].

Supplementary Table S2: Oligonucleotide primers used in this study

Gene		Sequence
**Plasmid construction**		
plemiR-30a	F:	5′-CCGCTCGAGTCCATGAGCCTTCTTA 3′
	R:	5′-CGACGCGTTATTGCCCTACTACGC 3′
pcDNA3/miR-30a	F:	5′-CCAAGCTTTCCATGAGCCTTCTTA 3′
	R:	5′-CGGAATTCTATTGCCCTACTACGC 3′
Slug 3′-UTR/wt	F:	5′-CCGCTCTAGATGGTTTGGTACTAATCATGAAG 3′
	R:	5′-TCCCAGTCTAGAACGCAATCAATGTTTACTCGAA 3′
Slug 3′-UTR/mut1	F:	5′-GTGACGCAATCAAACAAATGTCGAACAGAATGCATTTC 3′
	R:	5′-GAAATGCATTCTGTTCGACATTTGTTTGATTGCGTCAC 3′
Slug 3′-UTR/mut2	F:	5′-CAATGTAAATTTGCGGTGCTTTTTATATGATTG 3′
	R:	5′-CAATCATATAAAAAGCACCGCAAATTTACATTG 3′
**Chromatin IP**		
CLDN1 (+171/+189)	F:	5′-ATCCAGACTCCAGCGCCGCC 3′
	R:	5′-GACTCGCTCGGGCGCCCGCG 3′
CLDN2 (-803/-717)	F:	5′-TTCAAAGAGAGGCAGTGTAAAC 3′
	R:	5′-TGAGAACCTGTGGCTTCTCTACT 3′
CLDN3 (-462/-363)	F:	5′-ACCGCCCGGACTCAGGCTGAGCC 3′
	R:	5′-GACACGGCTTCTCTCTCCTCACC 3′

The primer sequences with mutated nucleotides underlined are the sequences of the mismatch primers used to generate the different Slug 3′-UTR mutants.
